# Expression of brain-derived neurotrophic factor and formation of migrasome increases in the glioma cells induced by the adipokinetic hormone

**DOI:** 10.1590/1806-9282.20231337

**Published:** 2024-05-20

**Authors:** Sibel Köktürk, Sibel Doğan, Cansu Eda Yılmaz, Yeliz Cetinkol, Oğuz Mutlu

**Affiliations:** 1 Istanbul University, Faculty of Medicine, Department of Histology and Embryology – İstanbul, Turkey.; 2 Istanbul University-Cerrahpasa, Cerrahpasa Faculty of Medicine, Department of Pathology – İstanbul, Turkey.; 3 Afyonkarahisar Health Sciences University, Faculty of Medicine, Department of Medical Microbiology – Afyonkarahisar, Turkey.; 4 Kocaeli University, Faculty of Medicine, Department of Pharmacology – İzmit, Turkey.

**Keywords:** Adipokinetic hormone, Brain-derived neurotrophic factor, Migrasome, Migration, Glioma

## Abstract

**OBJECTIVE:**

It has been previously shown that brain-derived neurotrophic factor is linked with various types of cancer. Brain-derived neurotrophic factor is found to be highly expressed in multiple human cancers and associated with tumor growth, invasion, and metastasis. Adipokinetic hormones are functionally related to the vertebrate glucagon, as they have similar functionalities that manage the nutrient-dependent secretion of these two hormones. Migrasomes are new organelles that contain numerous small vesicles, which aid in transmitting signals between the migrating cells. Therefore, the aim of this study was to investigate the effects of Anax imperator adipokinetic hormone on brain-derived neurotrophic factor expression and ultrastructure of cells in the C6 glioma cell line.

**METHODS:**

The rat C6 glioma cells were treated with concentrations of 5 and 10 Anax imperator adipokinetic hormone for 24 h. The effects of the Anax imperator adipokinetic hormone on the migrasome formation and brain-derived neurotrophic factor expression were analyzed using immunocytochemistry and transmission electron microscope.

**RESULTS:**

The rat C6 glioma cells of the 5 and 10 μM Anax imperator adipokinetic hormone groups showed significantly high expressions of brain-derived neurotrophic factor and migrasomes numbers, compared with the control group.

**CONCLUSION:**

A positive correlation was found between the brain-derived neurotrophic factor expression level and the formation of migrasome, which indicates that the increased expression of brain-derived neurotrophic factor and the number of migrasomes may be involved to metastasis of the rat C6 glioma cell line induced by the Anax imperator adipokinetic hormone. Therefore, the expression of brain-derived neurotrophic factor and migrasome formation may be promising targets for preventing tumor proliferation, invasion, and metastasis in glioma.

## INTRODUCTION

Glioblastoma multiforme (GBM) is the most aggressive primary brain cancer in adults. Despite extensive research has been performed for better understanding of the GBM biology, it remains an incurable and deadly disease^1^.

The increased expression of BDNF is closely associated with enhanced tumor invasion and metastasis. Previous studies have demonstrated that BDNF plays a role in growth, invasion, and metastasis in several types of tumors, such as gastric cancer, lung cancer, hepatocellular carcinoma, and ovarian and prostate cancers. The BDNF binds to tropomyosin-related kinase B (TrkB) receptor and triggers the production of pro-migratory, pro-survival, and anti-apoptotic proteins^2,3^.

The adipokinetic hormones (AKHs) play a crucial role in the energy mechanism of insects. They are responsible for regulating the mobilization of carbohydrates and lipids from the fat in insects^4^. The AKHs are functionally related to the vertebrate glucagon. The AKH-producing cells and pancreatic alpha cells share numerous similar mechanisms that manage the secretion of AKH and glucagon, respectively. The AKH release causes hyperglycemia through binding to the AKH receptor^5^. Hyperglycemia has been shown to induce proliferation in various cancer cells. Previous research has indicated a relationship between migration of cancer cells and glucose levels. The cancer cells increase glucose uptake to provide energy support to the proliferation and metastasis^6-8^.

In 2015, researchers discovered a new cellular mechanism and organelle, called migracytosis and migrasome^9^, which are a unique type of extracellular vesicles that help transmit signals between the migrating cells. The migrasomes are released into the environment and can be taken up by recipient cells. The migrasomes have been suggested to mediate intercellular communications, potentially causing physiological and pathological effects. The formation of migrasomes increases during cell migration^10^, which makes it a potential marker for detecting metastasis.

The aim of this study was to investigate the effects of Ani-AKH on BDNF expression and ultrastructure of cells in the rat C6 glioma cell line using immunocytochemistry and transmission electron microscope.

## METHODS

### Cell culture and treatments

The rat C6 glioma cell line used in this experiment was obtained from the American Type Culture Collection (ATCC-CRL2199). The cells were cultured for 24 h in a Dulbecco’s modified Eagle’s F12 cell culture medium with fetal bovine serum, penicillin, and streptomycin in addition to Ani-AKH. The cells were then seeded at a density of 1x10^5^ cells per well into 6-well plates with coverslips. Of note, 5 and 10 μM of Ani-AKH were added to the cells and incubated for 24 h. The numbers of living and dead cells were evaluated using trypan blue in the presence or absence of Ani-AKH (5 and 10 μM). No significant differences were observed between the number of living and dead cells in the Ani-AKH and control groups.

### Transmission electron microscopy

The culture cells were fixed with 2.5% glutaraldehyde in phosphate-buffered saline and then post-fixed with osmium-containing potassium ferrocyanide. The cells were then dehydrated in the graded ethanol and embedded in resin. After that, the ultrathin sections were cut and grids were stained with uranyl acetate and lead citrate. Finally, the samples were examined with a Jeol JEM 1011 transmission electron microscope (TEM).

### The brain-derived neurotrophic factor immunocytochemistry and immunofluorescence staining

The cells were evaluated using rabbit polyclonal anti-BDNF primary antibody (Santa Cruz Biotechnology, sc-20981, 1:50 dilution), ImmunoCruz ABC detection kit, and anti-goat IgG-fluorescein isothiocyanate (FITC) secondary antibody (Santa Cruz Biotechnology, K1715, 1:50 dilution). The coverslips were visualized using the Olympus BX61 fluorescence microscope with the DP72 Olympus camera system at 40x objective. Five digital images were randomly selected and analyzed using the Aperio ImageScope software. The BDNF positive pixel count algorithm was determined from five random views within the images. Comparisons between the groups were evaluated using a one-way ANOVA and Tukey post hoc tests by the KaleidaGraph 4.0 software. p<0.05 was considered significant.

## RESULTS

**Figure 1:**
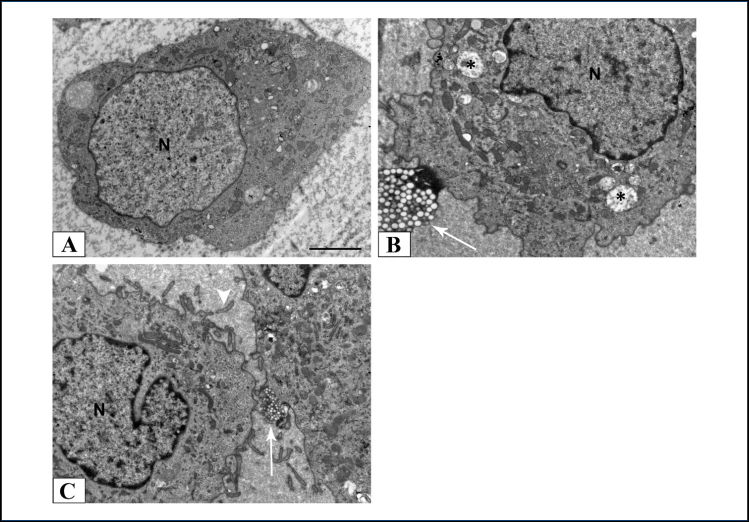
Transmission electron microscopic views of groups: control (A), Anax imperator adipokinetic hormone-5 (B), and Anax imperator adipokinetic hormone-10 (C). Visualization of migrasomes by transmission electron microscope [(B) and (C)]. The white arrow shows migrasomes connected with the cell body. N: nucleus; asterisk: vacuole; arrow: migrasome; arrowhead: filopodium. Scale bar=2 μm.

In the control group, C6 glioma cells had an oval shape with occasionally slightly indented euchromatin nucleus and a relatively smooth cell membrane with small and short filopodia and little blebs. The endoplasmic reticulum and mitochondria were distinguishable and small vacuoles appeared in some cells. However, the migrasome structures were not displayed in the C6 glioma cells of the control group. The alterations induced in C6 glioma cells of the Ani-AKH-10 group were similar to those observed in C6 glioma cells of the Ani-AKH-5 group. Both AKH groups were found to multiply cleft nucleus that was divided into several lobes of irregular sizes. Filopodia were noticed in their cell membranes, and their cytoplasm was rich in vacuoles. The C6 glioma cells of the Ani-AKH-5 and Ani-AKH-10 groups showed the membrane-bound vesicular structures with cytoplasmic extensions observed around the C6 glioma cells using TEM. These membrane structures, called migrasomes, contained numerous smaller vesicles and elongated toward other cells. Ani-AKH-5 and Ani-AKH-10 groups induced the migrasome formation without cytotoxicity (Figure 1).

The BDNF staining intensity of Ani-AKH-5 and Ani-AKH-10 groups were significantly higher than the control group (p<0.0001). The BDNF immunofluorescence staining results were consistent with that seen in the immunocytochemistry staining results. The immunofluorescence positive staining for BDNF was detected throughout the cytoplasm from the perinuclear region of rat C6 glioma cells (data not shown).

## DISCUSSION

Glioblastoma multiforme is the most lethal tumor of the central nervous system with limited treatment options. Standard GBM therapy includes neurosurgery, radiotherapy, and chemotherapy. However, despite these treatments, GBM remains a lethal tumor with a poor prognosis, with a median overall survival estimated between 15 and 17 months, and a 5% survival rate at 5 years^11,12^. The GBM cells communicate with the tumor microenvironment for their own benefit. The various communication ways in GBM cells lead to affect tumor growth, metastasis, and angiogenesis. Therefore, GBM cells make use of various communication ways to interact with the surrounding cells^13,14^.

Migrasome plays an important role in various fields including cell-to-cell communication, and occurrence, progress, and diagnosis of various diseases. The information transfer among cells may be the primary function of migrasomes^15^. Migrasomes are generated in a variety of cells, including metastatic tumor cells. In the cancer cells, production of migrasomes is significantly higher compared to normal cells, which increases their migration ability. Migrasome production depends on cell migration, and migrasome-mediated intercellular information transmission is extremely special because it only exists in migrating cells^16^. The regulation and functions of migrasome formation is not well understood^17^. Previous studies have suggested that migrasomes may have an important effect on the occurrence and development of cancer and might be new targets for cancer treatment. As a new target for disease treatment, the intervention of migrasomes may save patients’ lives and improve their prognosis. Blocking the migration-promoting effect of migrasomes through various factors may be a new strategy for anti-metastasis therapy. The studies on the relationship between migrasomes and diseases are still ongoing^18-20^. While one cell migrates away from its current place, it leaves migrasomes behind and then another cell takes these migrasomes^9^. During this study, it was often observed that migrasome left behind by one cell is taken up by another cell^21^. The migrasome-mediated communication between GBM cells and the tumor microenvironment may be associated with tumor progression and recurrence. One of the functions of migrasomes is probably cell–cell communication.

The rat C6 glioma cells were examined using TEM to control whether migrasomes are present. The migrasomes were observed as plasma membrane-bound vesicular structures in the extracellular space around the rat C6 glioma cells. These migrasomes were oval-shaped and contain various small vesicles.

The AKH is a neuropeptide hormone, which is a member of the gonadotropin-releasing hormone superfamily. The AKH-producing cells and human pancreatic alpha cells command many similar mechanisms controlling the secretion of AKH and glucagon^5,22^. While a previous study showed the stimulating effect of glucagon on the growth of human colorectal adenocarcinoma cells, the mechanism by which glucagon stimulates colon cancer cell proliferation remains uncertain^23^. Cancer cells require high glucose uptake to support cell survival, growth, and metastasis. The present study showed that Ani-AKH induced the formation of migrasome in the Ani-AKH-5 and Ani-AKH-10 groups.

Recently, the BDNF has been shown to be overexpressed in various types of cancer and associated with their poor prognosis in promoting tumorigenesis and progression^24^. The effect of BDNF on cellular functions is induced by receptor TrkB. The BDNF activates the AKT pathway in order to maintain cell survival^25^. Thus, the BDNF inhibits cell apoptosis and facilitates cell proliferation in various human cancers. In this study, the rat C6 glioma cells induced by Ani-AKH showed high expression of BDNF and formation of migrasomes in the Ani-AKH-5 and Ani-AKH-10 groups compared to the control group.

## CONCLUSION

Glioblastoma multiforme is the most lethal tumor of the central nervous system. Intercellular communication represent an essential feature for proliferation and metastasis^1^. Further comprehension of the complex mechanics taking place between migrasome formation, expression of BDNF, and metastasis could have a great beneficial influence on the therapeutic results of patients with GBM and may ensure the generation of new treatment strategies.

This study revealed that the C6 glioma cells induced by the Ani-AKH increased migrasome formation and expression of BDNF, and thus also increases the relationship between BDNF and migration. The C6 glioma cells have an increased BDNF expression. Increases of BDNF expression may have an impact on its ability to produce migrasomes. Our research has also provided a way for identification and to study the mechanism of migrasome formation. The findings from this study also suggest the possible use of the new therapeutic targets such as BDNF and formation of migrasome in the glioma treatment. Hence, there is a need to search for new strategies to decrease the BDNF level and formation of migrasome as a target to prevent metastases and discover therapy for various cancers such as glioma.

Only few clinical studies exist on migrasomes. The clinical use of migrasome formation and BDNF expression inhibitors in combination with standard chemo- or radiotherapy or other signal transduction pathway inhibitors may make a significant contribution to the treatment of GBM.
